# A Rapid Review of Interventions to Increase Hepatitis B Testing, Treatment, and Monitoring among Migrants Living in Australia

**DOI:** 10.3390/ijerph19105947

**Published:** 2022-05-13

**Authors:** Vishnupriya Rajkumar, Kahlia McCausland, Roanna Lobo

**Affiliations:** 1 School of Population Health, Curtin University, Bentley, WA 6102, Australia; kahlia.mccausland@curtin.edu.au (K.M.); roanna.lobo@curtin.edu.au (R.L.); 2 Collaboration for Evidence, Research and Impact in Public Health (CERIPH), School of Population Health, Curtin University, Bentley, WA 6102, Australia

**Keywords:** migrants, hepatitis B, testing, treatment, monitoring, high-income countries, review, intervention

## Abstract

Chronic hepatitis B (CHB) disproportionately affects migrants with low health literacy and help-seeking behaviour living in high-income countries. Evidence of effective interventions is required to increase hepatitis B (HBV) testing, treatment, and monitoring. Available evidence from Medline, Embase, Scopus, Google, and Google Scholar was identified, collated, and synthesised. Inclusion criteria included grey and peer-reviewed literature published in English between January 2012 and December 2021. Systematic reviews and meta-analyses were excluded. Seventeen peer-reviewed articles met the inclusion criteria. Most interventions were conducted at the individual level and were typically outreach testing initiatives. One study was conducted at a structural level. All studies were successful in encouraging HBV screening uptake, and 10 studies demonstrated effective linkage to care. Two studies showed evidence of monitoring participants post-intervention. Most interventions had more female than male participants. Interventions conducted across community and clinical-based settings had more participants engage in screening and/or linkage to care in community settings. Effective interventions to prevent HBV transmission and CHB-related morbidity and mortality were approaches that utilised linguistic-specific and culturally appropriate resources to successfully engage migrants. Community outreach programmes that educate participants about HBV transmission, screening, and treatment can promote community dialogue and understanding to reduce stigma and discrimination.

## 1. Introduction

Globally, mortality from chronic viral hepatitis (CVH) has surpassed that of human immunodeficiency virus (HIV), malaria, and tuberculosis combined, prompting significant concern [1]. While the burden of disease is mainly carried by low-resource countries in sub-Saharan Africa, South East Asia, and the Western Pacific regions where hepatitis B (HBV) is endemic, the epidemiological spread of HBV to industrialised countries is driven by migration [2]. HBV is the most prevalent blood-borne and sexually transmissible virus in Australia, and when left untreated, chronic hepatitis B (CHB) can lead to serious adverse health outcomes such as liver cirrhosis and cancer [3]. Additionally, deaths attributable to liver cancer have increased faster than deaths caused by any other cancer, with liver cancer being the sixth leading cause of cancer mortality in Australia [4]. HBV in Australia disproportionately affects residents born overseas in HBV-endemic areas, accounting for 61% of infections [5,6], and can be attributed to the lack of a universal vaccination programme prior to 2014, mother-to-child (vertical) transmission, and transmission from other close contacts with CHB in the first years of life [1,5,6]. The introduction of childhood HBV vaccination programmes both overseas and locally reflect a decreased prevalence in the under-19 age group; however, more than 90% of new cases of CHB in Australia occur in migrants [6]. Accordingly, it is estimated, based on current trends of migration, and treatment and vaccination uptake, that an estimated 245,640 people will be living with CHB in Australia by 2030 [3].

In 2016, the World Health Organization called for the elimination of viral hepatitis by 2030 as part of the agenda to achieve sustainable development [7]. Accordingly, Australia has endorsed this strategy and has set targets to increase the total proportion of people living with CHB who are diagnosed to 80% and increase the total proportion of people living with CHB receiving care to 50% by the end of 2022 [3]. Although this strategy will significantly reduce liver cancer and cirrhosis mortality in Australia, achieving these targets is challenging. In 2019, only an estimated 68.8% of people living with CHB in Australia were diagnosed, and 22.1% were engaged in care, highlighting the profound progress that needs to be made to achieve Australia’s targets [3,5].

Progress towards meeting the HBV diagnosis and treatment targets has been slow due to several factors. Firstly, a high proportion of people living with CHB in Australia are migrants from culturally and linguistically diverse (CALD) backgrounds who have experienced social marginalisation, hepatitis-related stigma in their new host community, and challenges in adapting to new environments and healthcare systems that have resulted in barriers to HBV testing, diagnosis, and care [8,9,10]. Furthermore, migrants from countries where HBV is endemic view the condition as expected and therefore normal, highlighting complacency around testing and management compliance [11]. Additionally, the low levels of HBV knowledge and awareness among migrants from CALD backgrounds are further propagated by the asymptomatic nature of the disease, resulting in low help-seeking behaviour [12]. Moreover, the COVID-19 pandemic has slowed progress toward HBV elimination targets due to disrupted routine vaccination campaigns and vaccine supply chains, altered transmission dynamics such as increased risk behaviours and disruption of harm reduction services, and decreased diagnostic and treatment services due to a diversion of resources [13].

Compared to people born in Australia, people born in countries with a high prevalence of HBV are six to twelve times more likely to be diagnosed with liver cancer due to CHB [14]. Improving early identification of HBV and enhancing linkage to care as well as treatment and preventive services for migrants from CALD backgrounds born in countries with an intermediate to high prevalence of HBV are urgently needed [2]. However, there is currently no national strategy to systematically screen those at risk for CHB in Australia, and many cases are only detected after the onset of irreversible complications [15].

Individual, community-based, and structural interventions have been fundamental in the prevention of HIV, CVH, and other sexually transmissible infections (STIs) globally and are considered to be critically important to engage individuals in testing and reduce barriers to healthcare access [16]. Interventions can be categorised by their level of action and approach to tackling health inequalities [17]. Individual interventions use person-centred strategies in a one-on-one setting, including education, advice, and counselling, to improve people’s knowledge, attitudes, skills, and behaviours, including uptake of testing and treatment adherence [17]. Community interventions seek to improve the health of disadvantaged communities by improving social cohesion and mutual support and strongly emphasise lessening social stigma through group-based health promotion, education, advice, and counselling [17,18]. Structural interventions, such as policy change, attempt to improve the social, physical, economic, and political contexts that influence the standard of living achieved by the whole population and their importance in treating HBV and other STIs [19,20,21,22].

Available reviews on HBV in migrant populations have highlighted disease prevalence, gaps in knowledge, health literacy levels, and experiences with healthcare providers [22,23,24] and have identified barriers to HBV testing, treatment, and monitoring faced by migrants from CALD backgrounds. This study conducted a rapid review of the available evidence on the effectiveness of interventions that aimed to increase HBV testing, treatment, and/or monitoring among migrants from CALD backgrounds living in Australia and other high-income countries: the United Kingdom (UK), Canada, New Zealand (NZ), and the United States (US). The findings are expected to assist efforts to reduce HBV-related morbidity and mortality in Western Australia, and Australia more broadly.

## 2. Materials and Methods

This review was conducted using the recommendations and minimum standards for rapid reviews by the Cochrane Rapid Reviews Methods Group and Preferred Reporting Items for Systemic Reviews and Meta-Analyses extension for Scoping Reviews guidelines (PRISMA-ScR) [23,25].

### 2.1. Search Strategy

The following search strategy was developed incorporating search terms used in similar reviews and in consultation with a university librarian: (“hepatitis B” OR (hep AND b) OR “hep* B” OR “viral hepatitis”) AND (interventions OR process* OR strateg* OR procedure OR screen* OR “mass screen*” OR diagnosis OR testing* OR monitoring OR target* OR care OR treatment) AND (CALD OR migrant* OR “culturally and linguistically diverse” OR transient OR overseas-born OR refugee OR humanitarian) AND (Australia* OR “New South Wales” OR Victoria OR Tasmania OR “West* Australia” OR “South Australia” OR Queensland OR “Australian Capital Territory” OR ACT OR “Northern Territory” OR NT OR “United Kingdom” OR UK OR Canada OR NZ OR “New Zealand” OR US OR USA OR “United States”).

### 2.2. Inclusion and Exclusion Criteria

To identify potentially relevant studies for inclusion, a comprehensive search of three databases and two search engines (Google and Google Scholar) was conducted. The first ten pages of search results from Google and Google Scholar were screened. The following academic databases were identified to be appropriate: Medline, Embase, and Scopus, as they contain references to journal articles in life sciences with a focus on biomedicine. The search strategy included both grey and peer-reviewed literature (quantitative or qualitative) published between January 2012 and December 2021 and in the English language. Articles characterised as systematic reviews and meta-analyses were ineligible for inclusion. Due to a lack of Australian studies focusing on HBV interventions to increase testing, treatment, and/or monitoring among migrants from CALD backgrounds, eligible studies included those conducted with first- and/or second-generation migrants from CALD backgrounds living in the UK, the US, NZ, and Canada.

This review defined a high-income country as one with a gross national income per capita of USD 12,696 or more [24]. A migrant was defined as “any person who changes his or her country of usual residence” and included migrant workers, international students, refugees, and asylum seekers [26]. A first-generation migrant was defined as foreign-born, and a second-generation migrant was defined as a person who was born and is residing in a country that at least one of their parents entered as a migrant [16]. Second-generation migrants were included in this study as unvaccinated household contacts of CHB-positive individuals are susceptible to HBV infection [6].

### 2.3. Screening

The primary researcher (V.R.) conducted the same searches in each database to ensure a complete, consistent, and comprehensive process. The citations identified through the search strategy were exported into Endnote X9 citation management software. Duplicate citations were removed before exporting the remaining citations into Rayyan, a systematic review management software where further duplicate citations were identified and removed [27,28].

In Rayyan, two reviewers (V.R./K.M.) screened the titles and abstracts of the imported citations independently of one another (a double screening approach) to determine eligibility, reducing the risk of bias and promoting the selection of the relevant literature [29]. For the citations that were identified as potentially eligible, or that required further investigation, the full text was retrieved to further assess eligibility and reviewed by V.R. Reasons for excluding studies during the full-text review process were studies reporting current HBV prevalence in migrants, barriers that migrants faced to accessing healthcare, and inclusion of other population groups, such as people who inject drugs. A third reviewer (R.L.) reviewed articles where eligibility was uncertain. The reference lists of the eligible literature were also screened to ensure no relevant studies were missed.

### 2.4. Data Extraction

The research team developed a pro forma data extraction table containing the following headings: Author(s), Year of publication, Location of study, Population and sample size, Aim, Methodology, Intervention type, Outcome measures/results, Implications for research, policy, and/or practice. The expanded data extraction table is available in the Appendix A. Two reviewers (V.R./K.M.) extracted data from five articles and then convened to compare the data extracted. This process served to assist the primary reviewer (V.R.) with consistency when extracting data from the remaining papers.

### 2.5. Evidence Synthesis

Relevant outcome measures identified from the literature were reported, which focus on aspects of interventions that are successful and unsuccessful, implications for research, policy, and practice, how these results relate to Australia, and strategies to increase HBV testing, treatment, and monitoring.

## 3. Results

**Figure 1:**
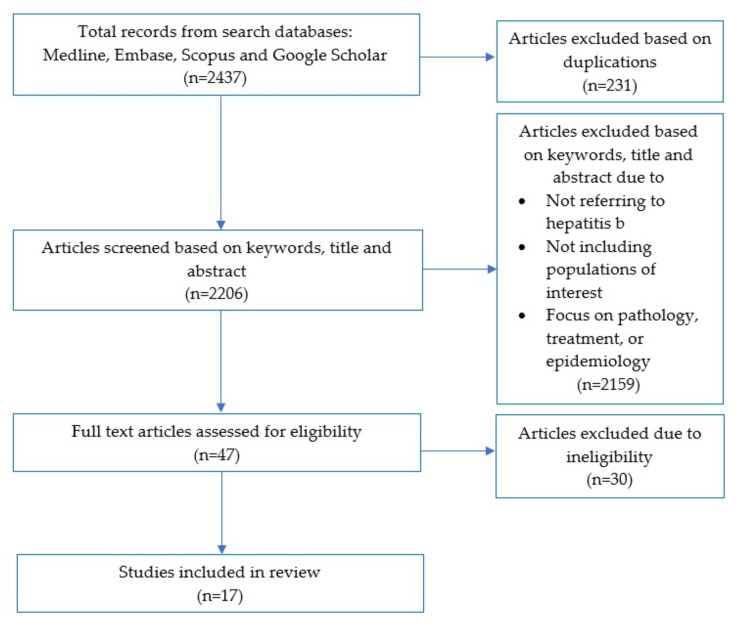
PRISMA flow diagram of the review process.

The screening process was documented and reported using a PRISMA flow diagram [30] (Figure 1). The search retrieved 2437 articles. After duplicates were removed, a total of 2206 articles were screened based on their keywords, title, and abstract, and from this, the full texts of 47 articles were assessed for eligibility. After full-text review, a further 30 articles were excluded as they measured HBV prevalence in the target group, or the study included other population groups such as persons injecting drugs. A final sample of 17 studies met the inclusion criteria.

The populated data extraction pro forma is available in Table 1.

### 3.1. Overview of the Studies

Of the 17 studies, 9 were conducted in the US [32,33,35,38,40,41,42,43,44], 5 in the UK [34,36,37,39,45], 2 in Australia [31,46], and 1 in Canada [47]. Five studies used mixed methods (qualitative and quantitative) [31,39,45,46,47], and the remaining twelve studies used quantitative methods [32,33,34,35,36,37,38,40,41,42,43,44]. The sample size of the studies ranged from 54 to 827 in the mixed methods studies [31,39,45,46,47], and from 96 to 90,250 participants in the quantitative studies [32,33,34,35,36,37,38,40,41,42,43,44]. All studies collected information between 2009 and 2020 [31,32,33,34,35,36,37,38,39,40,41,42,43,44,45,46,47]. One study did not report ethics approval from a human research ethics committee [42].

### 3.2. Participant Characteristics

Of the 17 studies, 2 studies examined migrants from South East Asia (SEA) [36,39], 3 focused on migrants from North East Asia (NEA) [36,47,48], 3 focused on migrants from both SEA and NEA [37,42,49], 2 focused on migrants from sub-Saharan Africa (SSA) [32,43], and the remaining 7 studies were on migrants from a mixture of SEA, NEA, SSA, and other regions [34,35,39,41,43,45,50]. All studies included both male and female participants. Thirteen studies had more female than male participants [32,33,35,36,38,39,40,41,42,44,45,46,47], and the remaining four studies had more male than female participants [40,43,45,50]. Participants’ ages varied among studies, with six including participants over 18 years of age [37,40,42,44,46,48], and the remaining studies including participants under 18 years of age [36,47,50] or not stating the lower limit of their participants’ age range [34,35,38,39,41,43,45,49]. A majority of studies included broad migrant communities, with some studies conducted with refugees [35,43,50] and asylum seekers [37]. Six studies explicitly stated that 71% to 100% of the participants had a preferred language other than English [35,36,39,42,48,49].

### 3.3. Individual Interventions

Individual interventions consisted of outreach testing and provider-initiated interventions such as testing and linkage to care (vaccination or referral to primary care or a specialist provider for treatment). Outreach testing refers to locating at-risk populations where they socialize, and provider-initiated interventions are defined as testing and counselling services recommended by health professionals as a standard component of medical care [48,50]. Of the 17 studies, 13 studies reported individual interventions [31,33,35,36,37,38,40,41,42,43,44,45,46], and they were categorised by the method(s) used to encourage testing and linkage to care.

#### 3.3.1. Outreach Testing

Outreach testing was conducted by seven studies in the US [33,35,40,41,42,43,44], one study in the UK [45], and one study in Australia [46]. Of these studies, outreach efforts were effective in encouraging testing in all studies, and eight studies identified HBV prevalence in their target populations ranging from 3.0% to 9.6% [33,35,40,41,42,43,44,45].

Six studies showed evidence of linkage to care ranging from 28% to 97% of the participants, such as counselling to receive HBV vaccination or referral to a primary care or specialist provider [33,35,40,41,43,44]. Three studies reported that males were more likely to test positive for HBV [36,40,42], and one study reported that women were five times more likely to have a previous HBV diagnosis compared to their male counterparts [45]. Navarro et al. [40] reported a discrepancy between serological results and self-declared vaccination history; out of 240 participants thought to have been previously vaccinated, only 60% (*n* = 145) had serological evidence of this (*p* < 0.001). No studies conducting outreach testing showed evidence of monitoring participants after they had been linked to care.

#### 3.3.2. Overview of Outreach Testing Strategies Utilised

All studies used linguistic-specific and culturally appropriate materials such as advertisements in local newspapers and announcements on radio stations in languages used by local migrant communities or bilingual/multilingual patient navigators, including community and religious leaders, to recruit and communicate with their target populations [33,35,40,41,42,43,44,45,46]. Seven studies provided education on HBV in conjunction with screening events [33,41,42,43,44,45,46]. Six studies conducted interventions in community-based settings (public schools, places of worship, or community centres) [35,40,41,43,45,46], and three studies conducted interventions across both community (health fairs or faith-based organisations) and clinical-based settings (primary care or emergency departments) [34,35,39].

Of the interventions conducted across community and clinical-based settings, the studies by Chandrasekar et al. [33] and Raines-Milenkov et al. [42] reported that more participants engaged in testing in community settings compared to clinical-based settings, and the former linked more participants tested in community settings to medical care. The study by Dang and Chen Jr. [35] reported that 72% (*n* = 21) of the participants eligible for vaccination completed the series of doses required for full immunisation. Other studies by Perumalsawmi et al. [41] and Shankar et al. [43] also reported that 37% and 57% of the participants were eligible for HBV vaccination, respectively. However, there was no information on post-intervention vaccination rates in either study, even though Perumalswami et al. [41] offered free HBV vaccines at screening sites. Shankar et al. [43] offered HBV-positive participants cash and travel-cost incentives after the completion of follow-up visits, and Standford et al. [44] offered participants cash incentives after they completed HBV testing. One study used dried blood spot sampling to test for HBV, which offered advantages such as a reduced risk of needle-stick injury, safer transportation of samples, and the ease of storing samples at room temperature [45]. A study by Xiao et al. [46] compared the impact of educational resources on HBV testing uptake. Participants were randomised to receive either standard HBV-related information or education focusing on liver cancer prevention, and at the end of the study, the latter group had an HBV testing uptake four times that of the former [46].

### 3.4. Provider-Initiated Testing

Provider-initiated testing was conducted by Flanagan et al. [36] and Hargreaves et al. [37] in the UK, Hsu et al. [38] in the US, and Ash et al. [31] in Australia. Three interventions among these studies were effective in encouraging up to 90% of their target population to engage in HBV screening [37,44,50]. Ash et al. [31] identified and immunised 6% of uninfected susceptible individuals in their target population but did not find any new cases of CHB. Studies by Flanagan et al. [36] and Hsu et al. [38] linked 80% and 50% of the HBsAg-positive participants to care, respectively. Only one study [38] monitored HBsAg-positive or HBV-non-immune individuals for three months post-intervention.

#### Overview of Provider-Initiated Strategies Utilised

All studies except for Flanagan et al. [36] used linguistic-specific and culturally appropriate materials to communicate with their target populations. All studies were conducted in a clinical-based setting [37,44,45,50]. Flanagan et al. [36] provided participating general practitioners with education, a financial incentive, clinician support, and electronic prompts when accessing patient records to screen migrants for CVH. Although the intervention was successful compared to the control (20% vs. 2%), it had a lower than expected screening uptake and fell short of the assumption that at least 40% of the eligible participants would be screened. A similar intervention by Hsu et al. [38] that used electronic health record prompts tracked HBV-positive participants for three months post-intervention and vaccinated 25% of susceptible individuals. In a “one stop” screening initiative in a UK hospital emergency department, Hargreaves et al. [37] found that while 61% of patients presenting to the service were reported to be from an ethnic background, it was not possible to ascertain the number of migrants in this group that did not consent to participate in the study. The nurse-led contact tracing system by Ash et al. [31] yielded 420 contacts from 122 CHB-positive index patients and vaccinated 27 susceptible individuals.

### 3.5. Community Interventions

Community interventions consisted of group or peer education initiatives. A total of 3 of the 17 studies conducted community interventions to address low levels of HBV knowledge, awareness, and associated stigma [38,46,49]. All three studies were effective in encouraging participants to consent to HBV testing, ranging from 19% to 84% of their target populations [38,46,49]. In one of the studies, four participants (1%) requested a HBV vaccination within four weeks of attending a community education workshop [47].

#### Overview of Community Intervention Strategies Utilised

All studies used linguistic-specific and culturally relevant methods to communicate with their target populations [38,46,49]. Two studies conducted interventions across community and clinical-based settings [32,39], or in community settings only [47]. In addition to screening and linkage to care, the education workshops conducted by Zibrik et al. [47] prompted participants to proactively read information online or in print (60%), talk to their doctor about HBV testing (48%), schedule an appointment with their doctor (7%), or check their vaccination status or that of a family member (6%). Word-of-mouth from participants who watched an educational film in a study by Kelly et al. [39] encouraged an additional 45 participants to engage in CVH screening. Dried blood spot sampling was used to test for HBV, and primary care recruitment was poorer compared to that in the community [39]. Chandrasekar et al. [32] employed a chain-referral sampling method to engage hard-to-reach individuals through their natural social networks and found that individuals without healthcare insurance were more comfortable presenting to a community setting. The same study also reported fear of reprisal as a reason that migrants were reluctant to utilise the chain referral system [32].

### 3.6. Structural Interventions

Structural interventions included interventions addressing broader social, economic, and political environments. Only 1 of the 17 studies in this review reported the results of a mandatory screening programme conducted in clinical-based settings [34]. The intervention screened a large cohort of UK-bound refugees as part of a mandatory pre-migration resettlement programme, and the testing yield for HBV was notably high at 2.0% (*n* = 188) and ranged by nationality from 0.6% for Iraq and 13% for South Sudan [34]. This study cited fear of legal implications affecting migrants’ rights to resettlement as a barrier to the self-reporting of risk factors for infectious diseases [34].

### 3.7. Study Recommendations

All included studies made various recommendations for clinical practice, health education and promotion, research, and policy. Nine studies supported the need for future interventions that address common barriers faced by migrants for HBV testing, diagnosis, and care by utilising culturally tailored education and collaboration with community groups [34,35,38,39,41,42,46,48,49]. Two studies highlighted the importance of a targeted approach to screening and linkage to care for at-risk groups such as prevalence linked to patients’ country of origin [43,45], and Hargraves et al. [37] recommended routine testing for HBV for new entrants from high-incidence countries in both community and clinical-based settings. Ash et al. [31], Flanagan et al. [36], Hsu et al. [38], and Navarro et al. [40] made recommendations related to improvements in clinical practice such as a nurse-led approach to contact tracing, incentives for clinicians to screen patients, and provider recommendations for testing due to their influence on increasing patient testing and treatment outcomes. One study provided policy recommendations and acknowledged the effectiveness of mandatory screening of refugees as part of a resettlement programme in identifying infectious diseases [34].

## 4. Discussion

The purpose of this rapid review was to identify, collate, and synthesise the literature pertaining to interventions that aim to increase HBV testing, treatment, and/or monitoring among migrants from CALD backgrounds living in Australia, the UK, Canada, NZ, and the US. In summary, 17 peer-reviewed journal articles published between 2012 and 2021 met the inclusion criteria for review. This review identified three levels of interventions (individual, community, and structural) and two key settings where interventions took place, namely, community, and clinical-based health services (primary care and other clinical health services).

### 4.1. Testing

All interventions were successful in encouraging HBV screening uptake among their target group/s of first- and/or second-generation migrants [31,32,33,34,35,36,37,38,39,40,41,42,43,44,45,46,47]. According to previously defined HBV endemicity levels, a prevalence of less than 2% is considered low, a prevalence of 2–4.99% is lower-intermediate, a prevalence of 5–7.99% is considered higher intermediate, and a prevalence of >8% is high [49]. Twelve studies in this review identified an HBV prevalence of more than 2% in their target populations, confirming the intermediate to high prevalence of HBV in these at-risk groups, highlighting the importance of targeted testing [32,33,34,35,37,38,40,41,42,43,44,45]. Three studies in this review reported an HBV prevalence of more than 8%, and the populations in these studies comprised African-born migrants and migrants of Chinese or Vietnamese origin [37,40,47]. This finding is consistent with reports stating that the regions with the highest HBV prevalence are SSA and East Asia [7,51]. Reported discrepancies between self-declared vaccination history and serological results post-testing could be due to recall bias, poor English proficiency and health literacy, confusion between hepatitis B and hepatitis C, lower education level attained, access to care, or a general lack of awareness of prior screening and/or healthcare [52,53]. This further highlights the importance of complete serological testing in migrants before recommending treatment options [40].

### 4.2. Treatment and Monitoring

Ten studies had effective linkage to care [31,33,35,36,38,40,41,43,44,47]. Two studies monitored participants post-intervention [38,47]. Given that all individuals living with CHB should be regularly monitored and their HBV viral loads assessed annually to monitor disease activity and inform treatment, it was expected that more interventions would have monitored participants linked to care for at least twelve months post-intervention [54]. Given the critical importance of vaccinating susceptible individuals against HBV [6], it was surprising that none of the studies that recommended vaccination to participants based on serological results [41,43] provided evidence of vaccination uptake.

### 4.3. Impact of Intervention Settings

Three out of the four studies [35,38,39,46] that conducted their intervention across community and clinical-based settings reported higher engagement of participants in a community setting with regard to HBV screening and/or linkage to care [35,39,46]. This was an expected finding as migrant populations from CALD backgrounds often draw on their social networks for support with regard to health-literacy-related tasks such as interacting with health professionals and making health-related decisions [55]. Furthermore, migrants view a community-based screening service favourably as it delivers “services to the people where they are” [18] (p. 7), and participants feel more comfortable in a familiar environment with staff that are likely to have been recruited from the community itself [18].

### 4.4. Role of Linguistic-Specific and Culturally Appropriate Resources

Culturally relevant interventions that incorporate appropriate language and culturally sensitive settings are essential when attempting to successfully engage migrants from CALD backgrounds [18]. The screening intervention by Flanagan et al. [36] fell short of its target by more than 50% despite providing electronic health record prompts to general practitioners. One explanation could be that this study did not employ linguistic-specific methods or culturally competent health workers to communicate with their target group. Language barriers have been identified as a pertinent issue for migrants regardless of their level of education or length of residence in a predominantly English-speaking country [18,55]. A study by Hyun et al. [55] on barriers to HBV health literacy faced by Korean Americans revealed that while some individuals can communicate in simple English during everyday tasks, they encountered difficulty when it came to describing their health concerns or understanding health advice.

### 4.5. Uptake of Intervention Based on Gender

The majority of the interventions in this review had more female than male participants. Three studies reported that females were more likely to test positive for HBV [36,40,42], and Vedio et al. [45] highlighted that men were five times more likely to have a previous HBV diagnosis at the time of screening. The findings mentioned above are likely due to gender differences in beliefs about health among migrant communities. Males tend to avoid consciously thinking about their health and define being healthy as not seeking medical help [56]. By contrast, females are more likely to be aware of their family medical history and make associated changes to their lifestyles [56]. Furthermore, females reported help seeking as one of their common behavioural responses for a range of symptoms and bodily changes [56]. The higher HBV prevalence in male migrants may also be attributable to differences in their social and sexual behaviours [57].

### 4.6. Study Design and Reporting Limitations

It was difficult to compare the effectiveness of the interventions due to the varying study designs adopted by the studies in this rapid review. Studies that used a randomised controlled trial design had clear differences in outcomes between the control and intervention groups. Conversely, studies that conducted a feasibility trial or observational study measured the uptake of intervention outcomes among their target group. Therefore, future scoping or systematic reviews on this topic could limit the study design to one type in their eligibility criteria to allow comparisons of study effectiveness.

### 4.7. Strengths and Limitations

This rapid review had several strengths. Firstly, multiple researchers reviewed the database search results and adopted a team approach to minimise errors and ensure the quality of studies retrieved during the search. This is consistent with the Cochrane Methodological Expectations of Cochrane Intervention Reviews guidelines for rapid reviews [23]. Additionally, this review only used studies published in the last ten years, ensuring that the information collected included current research findings, innovations in care, and recent trends in population outcomes and was in line with the current social and political contexts of care provided. Furthermore, this review only included outcome-driven studies, allowing the authors to assess the effectiveness of the interventions. The three levels of interventions conducted in the two key settings successfully achieved at least one intervention outcome (testing, treatment, and/or monitoring), better guiding future policy development, research, clinical practice, and health promotion initiatives.

Studies from the US accounted for just over half of the studies included in this review. Compared to the other countries analysed in this rapid review with universal health insurance programmes, only the US has a voluntary, private employer- and individual-based system with private insurance as the primary form of health insurance [58]. It is acknowledged that the results from the US studies may reflect the organisation of its health system. For example, individuals that do not have health insurance may be reluctant to participate in interventions due to their inability to pay for healthcare services. This review also excluded grey and peer-reviewed literature not published in English and acknowledges that studies in languages other than English could have provided valuable information to understand successful interventions for migrants from CALD backgrounds. Studies conducted in low-income countries may also have offered a wealth of information to provide additional context to the findings. There was also no risk of bias assessment conducted.

### 4.8. Implications for Research, Policy, and Practice

The recommendations of the studies included in this review mainly addressed challenges at the individual level. Emphasis was placed on methods to identify at-risk groups in both community and clinical-based settings and break down language and cultural barriers to improve healthcare engagement. Most of the studies focused on screening and treatment, possibly due to the complexity, time, and resources required for interventions that monitor participants after they are linked to care. The following sections provide implications for research, policy, health promotion, and clinical practice considering the broader literature.

#### 4.8.1. Research Opportunities

Reasons for the low participation of male migrants were not explored and warrant further investigation, and the limited number of studies that monitored HBV-positive participants post-intervention merits further research to explore patient retention after they are linked to care and the reasons why they may have ceased treatment to prevent associated morbidity and mortality.

Only two studies in this review were conducted in Australia [31,46]. While there are useful lessons learnt from the other studies in this review, it would be beneficial to test interventions in the Australian healthcare context to increase migrants’ participation in HBV screening and clinical management.

Despite the effectiveness of all studies in achieving at least one intervention outcome, each intervention worked at one of three levels of action (individual, community, or structural). A majority (*n* = 12) of the interventions in this review focused on changing individual behaviours in a one-to-one setting [31,33,35,36,37,38,40,41,42,43,44,45]. As individual interventions target short-term behaviour change, there is a growing consensus that prevention programmes for infectious diseases need to address the broader social structural context to bring about sustained behaviour change [16]. This is especially the case for groups that experience inequality, discrimination, and exclusion [16,59]. Therefore, future interventions should include the simultaneous use of evidence-based individual, community, and structural strategies—an approach that is consistent with the foundations of the Ottawa Charter for Health Promotion [60,61].

#### 4.8.2. Clinical Practice Opportunities

Primary care providers, including general practitioners and primary care nurses, are critical in identifying risk factors for chronic disease and implementing clinical management with early detection, follow-up, and monitoring of CHB [62,63]. Furthermore, it is mainly through primary care services that Australian patients are linked to specialist care [54]. Primary care services may vary widely in their provision of interpretation services which can result in patients either relying on family members as interpreters during healthcare appointments, and crucial information being omitted by family members if the consultation involves sensitive topics or specialist terminology, or patients seeking traditional medicine practitioners who speak their preferred language [64]. Therefore, interpreters should be recognised as an essential component of clinical services provided to migrants from CALD backgrounds, and HBV education and awareness materials should be available in multiple languages [11,44]. In this review, provider-initiated interventions with electronic health record prompts and incentives were successful in encouraging screening and linkage to care [36,38]. To further boost screening initiatives, medical practitioners and other healthcare workers should receive continuing education to aid in identifying persons at risk and linking them to screening [63].

#### 4.8.3. Health Promotion Opportunities

Studies within this review stressed the importance of an ongoing need for community outreach programmes that build awareness and educate vulnerable populations about HBV to promote prevention, screening, and treatment. The key features of programmes should correct culturally rooted myths and conceptions about HBV transmission and educate participants on long-term risks and consequences to health [65]. Consultation and collaboration with relevant stakeholders, including community and religious leaders, should be considered to promote community dialogue, and understanding to reduce stigma and discrimination [15,66]. Additionally, effective health education should incorporate understanding and navigation of the healthcare system to connect patients with primary care resources and encourage patient-physician dialogue [66]. Furthermore, for any education and awareness programme to be effective, it must be culturally tailored to migrants from CALD backgrounds [67]. Therefore, outreach initiatives should involve facilitators from similar cultural backgrounds to participants, and educational resources should be developed in participants’ native language(s) [67,68].

#### 4.8.4. Policy and Advocacy Opportunities

Currently, there are no pre-arrival immunisation requirements for migrants or refugees entering Australia [69]. Although the Australian Immunisation Handbook advises the targeted catch-up vaccination of all migrants and refugees without valid vaccination documentation, no national system exists to achieve this [69]. Therefore, a universal tool for recording past immunisations is recommended, enabling primary care providers to assess immunisation needs among migrants and refugees [66].

Another barrier identified by Crawshaw et al. [34] and Chandrasekar et al. [32] to the self-reported history or voluntary screening of STIs identified by migrants is the fear of reprisal or legal implications affecting their rights to resettlement. Accordingly, it should be emphasised that neither mandatory screening nor catch-up vaccination will be used as a process of exclusion for those who test positive [70].

## 5. Conclusions

Global migration has resulted in an increasing number of CHB infections in high-income countries. Although Australia has endorsed the World Health Organization’s strategy to eliminate viral hepatitis by 2030 and has set targets to increase testing and linkage to care, effective and coordinated responses for migrants from CALD backgrounds have been slow, with limited reporting of Australian interventions in the literature. To prevent further HBV transmission and CHB-related morbidity and mortality, there is a critical need for public health approaches that consider broader socioeconomic and sociocultural factors associated with migrants from CALD backgrounds. Based on this review, interventions should utilise linguistic-specific and culturally appropriate resources to successfully engage migrants from CALD backgrounds, and community outreach programmes should educate participants to promote community dialogue and understanding to reduce stigma and discrimination.

## Figures and Tables

**Table 1:** Populated data extraction pro forma.

Author and Publication Date	Title	Population	Sample Size (*n*) and Gender (Female/Male)	Location	Intervention	Intervention Type	Setting	Outcome Measures	Population Screened	HBV Prevalence	Linkage to Care	Monitoring
Ash et al. (2018) [31]	Hepatitis B contact tracing: what works?	Refugees from high-prevalence countries	122 (41% female, 59% male)	Australia	Auditing of clinical records and contact tracing (CT)	Individual	Primary care	Proportion of contacts traced and serologically confirmed as infected, immune, or susceptible to HBV, enablers of CT success	N/A	N/A	6.4% of total participants in the intervention	N/A
Chandrasekar et al. (2016) [32]	A novel strategy to increase identification of African-born people with chronic hepatitis B virus infection in the Chicago metropolitan area, 2012–2014	African-born persons	1000 (51% female, 49% male of 445 screened)	US	Education and chain-referral sampling by community health workers	Community	Community (church groups) and primary care	HBV testing uptake and linkage to care uptake	45.0%	8.0%	N/A	N/A
Chandrasekar et al. (2015) [33]	A comparison of the effectiveness of hepatitis B screening and linkage to care among foreign-born populations in clinical and non-clinical settings	Foreign-born Asian Americans	758 (62% female, 38% male)	US	Educational and outreach intervention using bilingual community health workers	Individual	Community (health fairs organised by churches and social services) and primary care	HBV screening uptake and linkage to care uptake	N/A	7.3%	55.0% (clinical), 77.0% (non-clinical)	N/A
Crawshaw et al. (2018) [34]	Infectious disease testing of UK-bound refugees: A population-based, cross-sectional study	Foreign-born refugees	18,418 (48.8% female, 51.2% male)	UK	Pre-entry health assessment	Structural	Clinical health service	HBV screening	N/A	2.0%	N/A	N/A
Dang et al. (2016) [35]	Increasing hepatitis B testing and linkage to care of foreign-born Asians, Sacramento, California, 2012–2013	Foreign-born Chinese, Hmong, Korean, and Vietnamese	1004 (62.7% female, 37.7% male)	US	Outreach testing	Individual	Community (student-run medical clinics, heritage associations, and churches)	HBV screening uptake	N/A	7.6%	67.1%	N/A
Flanagan et al. (2019) [36]	Case finding and therapy for chronic viral hepatitis in primary care (HepFREE): A cluster: randomised controlled trial	Migrants from high-risk countries	90,250 (52% female, 48% male)	UK	Incentivising GPs	Individual	Primary care (general practices)	HBV screening uptake	19.5% (intervention group)	1.1%	>80.0% of HBsAg-positive participants	N/A
Hargreaves et al. (2020) [37]	Delivering multi-disease screening to migrants for latent TB and blood-borne T viruses in an emergency department setting: A feasibility study	Foreign-born economic migrants, asylum seekers, and refugees	96 (48% female, 52% male)	UK	Opportunistic screening	Individual	Other clinical health service-based (emergency department)	HBV screening uptake	N/A	2.0%	N/A	N/A
Hsu et al. (2013) [38]	Electronic messages increase hepatitis B screening in at-risk Asian American patients: A randomized, controlled trial	Chinese and Vietnamese patients	175 (61.4% female, 38.6% male in intervention group)	US	Electronic health record (EHR) prompts	Individual	Primary care	Ordering of HBV tests and linkage to care uptake	40.9%	13.3%	50.0% of HBsAg+ (referred to specialists), 25.0% of susceptible vaccinated for HBV	Monitored HBsAg-positive or HBV-non-immune for three months post-intervention
Kelly et al. (2020) [39]	Improving uptake of hepatitis B and hepatitis C testing in South Asian migrants in community and faith settings using educational interventions—A prospective descriptive study	South Asian migrants	219 (53% female, 47% male)	UK	Educational film	Community	Community, religious centres, and primary care	CVH testing uptake	84.0%	0.9%	N/A	N/A
Navarro et al. (2014) [40]	Lower than expected hepatitis B virus infection prevalence among first-generation Koreans in the US: Results of HBV screening in the Southern Californian Empire	First-generation Koreans	1007 (60.5% female, 39.5% male)	US	Educational and outreach intervention led by a bilingual nurse	Individual	Community (churches)	HBV screening uptake and linkage to care uptake	N/A	3.0%	27.5%	N/A
Perumalswami et al. (2013) [41]	Hepatitis Outreach Network: A practical strategy for hepatitis screening with linkage to care in foreign-born communities	Foreign-born from countries with a high prevalence of CVH	1603 (50.6% female, 49.4% male)	US	Educational and outreach intervention	Individual	Community (public schools, places of worship, YMCAs, public parks, hotels, business centres, and train stations)	HBV and HCV screening and linkage to care uptake	N/A	4.7%	57.0%	N/A
Raines-Milenkov et al. (2021) [42]	Hepatitis B virus awareness, infection, and screening multiethnic community intervention for foreign-born populations	Refugee immigrant population	1069 (80% female, 20% male)	US	Educational and outreach intervention by bicultural community health workers	Individual	Community (churches, mosques, civic groups) and primary care	HBV screening uptake	38.0%	6.0%	N/A	N/A
Shankar et al. (2016) [43]	A novel collaborative community-based hepatitis B screening and linkage to care program for African immigrants	African-born persons	955 (24.5% female, 75.5% male)	US	Outreach testing and linkage to care with a culturally targeted patient navigator	Individual	Community (community centres, places of worship, and sites of employment)	HBV screening and linkage to care uptake	N/A	9.6%	97.0%	N/A
Standford et al. (2016) [44]	Community-engaged strategies to promote hepatitis B testing and linkage to care in immigrants of Florida	Foreign-born nationals	1516 (50.4% female, 49.6% male)	US	Educational and outreach intervention by community health workers and certified medical assistants	Individual	Community (faith-based organisations, refugee servicing organisations), primary care, and the emergency department	HBV screening uptake and linkage to care uptake	N/A	4.4%	63.0%	N/A
Vedio et al. (2013) [45]	Hepatitis B: Report of prevalence and access to healthcare among Chinese residents in Sheffield, UK	Chinese residents	229 (57.5 female, 42.5% male)	UK	Educational and outreach intervention	Individual	Community (Kinhon Chinese Centre)	HBV screening uptake and participant feedback	N/A	8.7%	N/A	N/A
Xiao et al. (2021) [46]	Assessing the feasibility, acceptability and impacts of an education program on hepatitis B testing uptake among ethnic Chinese in Australia: results of a randomised controlled pilot study	Individuals of Chinese ethnicity	54 (69% female, 31% male)	Australia	Educational and outreach intervention	Individual	Community-based organisations	HBV testing uptake, intervention acceptability, and feasibility of the study	12%	N/A	N/A	N/A
Zibrik et al. (2018) [47]	Let’s Talk About B: Barriers to hepatitis B screening and vaccination among Asian and South Asian immigrants in British Columbia	Korean, Chinese, Filipino, and South Asian immigrants	827 (70% female, 30% male)	Canada	Culturally tailored education workshops	Community	Community (community centres, immigration settlement service centres, organised health events, and religious/cultural gathering places)	Self-reported action related to HBV prevention and management	19.0%	N/A	1.0%	Participant feedback was obtained two weeks and one month post-intervention

## Appendix A

**Author and Publication Date**	**Title**	**Population and Sample Size**	**Location**	**Aim**	**Methodology**	**Intervention, Intervention Type and Setting**	**Outcome Measures**	**Results**	**Implications for Research, Policy and Practice**
Ash et al. (2018) [31]	Hepatitis B contact tracing: What works?	Refugees from high-prevalence countries. *n* = 122 (41% female, 59% male)	Australia	To describe the structure of the contact tracing system; and to determine its effectiveness and identify enablers of success.	A retrospective clinical audit conducted over 2 months. Index cases were CHB-positive patients identified in the practice’s Chronic Disease Management (CDM) Care Plan database. Contacts were individuals listed as household contacts on a ‘contact tracing chart’ by each index case. Interviews were conducted with health professionals across different disciplines to identify enablers of contact tracing (CT) success.	Auditing of clinical records and CT; Individual; Primary care.	Proportion of contacts traced and serologically confirmed as infected, immune or susceptible to HBV, enablers of CT success.	There were 122 index cases and 420 contacts. 339 (80.7%) contacts were immune (121 because of cleared previous HBV infection and 218 through prior vaccination). 34 (8.1%) were already identified as CHB-positive through new arrival screening. 90.5% of contacts were either immune or infected and would not benefit from subsequent vaccination. 379 (90.2%) contacts were already registered at the practice. 380 (90%) contacts were successfully traced. In total, 83 (68%) of index cases had all their contacts successfully traced. CT enabled the identification and immunisation of 27 (6.4%) uninfected susceptible individuals.	CT system used serological evidence, which is likely to be more accurate as it does not rely on patient recall or knowledge of contact tracing. It also does not rely on clinician reports, which may vary depending on individuals’ definitions of successful contact, which can overestimate the success. CT system provided an organised and convenient system of reference in the case of enquiry of immune status. Therefore, if household members changed over time, the CT system ensured a systematic approach to reviewing and updating household contacts and inviting new household members for screening. Integration into the CDM Care Plan scheme provides time for CT, patient education and administration.
Chandrasekar et al. (2016) [32]	A novel strategy to increase identification of African-born people with chronic hepatitis B virus infection in the Chicago metropolitan area, 2012–2014	African-born persons. *n* = 1000 (51% female, 49% male of 445 screened)	US	To use a novel strategy of chain referral sampling to conduct HBV testing and describe the prevalence of chronic HBV infection among a sample of people born in Africa and now residing in the Chicago Metropolitan area.	A hepatitis education and prevention program was developed in collaboration with academic, clinical, and community partners for immigrant and refugee populations at risk for HBV infection. Community health workers implemented chain referral sampling, a novel strategy for recruiting hard-to-reach participants, targeting African-born participants. Participants were tested in both clinical and non-clinical settings. The clinic settings included community and federally qualified health centres, charitable organizations, solo and small group physician practices, and local hospitals. Physicians briefly educated participants about HBV and offered a free blood test. Non-clinical settings consisted of health fairs and other events hosted by community and faith-based organizations. Screenings followed educational workshops delivered in the target languages of participants or interpreted by trained bilingual community health workers.	Education and chain-referral sampling by community health workers; Community; Community (church groups) and primary care.	HBV testing uptake and linkage to care uptake.	Tested 45% of the target population and identified 8% with chronic HBV infection. The strategy leveraged community health workers, in conjunction with peer and faith-based leaders, who were in the position to engage a hard-to-reach population. Intervention reached a population who needed services but were limited by access and language barriers. Chain referral sampling helped to overcome some challenges of limited English language comprehension, illiteracy, and mistrust. Additionally, the strategy led to the identification of people susceptible to HBV infection and facilitated HBV vaccination.	There is a need for health promotion programs that are culturally appropriate and community-driven. Cultural barriers including denial and social stigma, as well as concerns about the cost of treatment if tested positive, tended to perpetuate racial/ethnic disparities in HBV disease despite the involvement of peers. The recruitment strategy helped to mitigate this distrust because of the natural social networks that contextualized participants’ involvement. Recommendation for HBV testing by respected fellow members of the community promoted buy-in.
Chandrasekar et al. (2015) [33]	A comparison of effectiveness of hepatitis B screening and linkage to care among foreign-born populations in clinical and non-clinical settings	Foreign-born Asian Americans, *n* = 758 (62% female, 38% male)	US	To demonstrate the comparabilityof screening participation and follow-up linkage to care forthe chronically infected in clinical and non-clinical settings.	The Asian Health Coalition (AHC) and its community partners conducted HBV screenings and follow-up linkage to care in both clinical and non-clinical settings. Participants were notified of their test outcome by letter if immune, if susceptible and requiring vaccination or if chronically infected and provided with instructions for seeking medical attention. Screenings in non-clinical settings were held in conjunction with an education session on HBV delivered either in the target language of the community or with interpretation using bilingual community health workers trained by AHC staff in the Hepatitis Education and Prevention Program (HEPP) model. Participants were offered free HBV screenings after the education sessions.	Education and chain-referral sampling by community health workers; Community; Community (church groups) and primary care.	HBV testing uptake and linkage to care uptake.	Prevalence of HBsAg in the sample was 7.3% (*n* = 55). Individuals who ended up testing positive for the disease were as likely to participate in clinical and non-clinical settings. Of those who tested positive in clinical settings, 9 of 16 (55%) were referred to follow-up medical care. For those tested in a non-clinical setting, 30 out of 39 (77%) were referred to medical care. This shows a higher linkage to care in non-clinical settings.	Demonstrates the merits of using a community-based model for linkage to care. HBV screenings for Asian Americans in non-clinical settings were valued equally to the clinical settings. Findings showed that there were no significant differences. This outcome is consistent with the hypothesis that non-clinical settings can reach a large segment of underserved foreign-born populations who tend to be uninsured and have limited English proficiency. A significant finding was the successful linkage to care available in non-clinical settings as compared to clinical settings. Community health workers in non-clinical settings are also integral pillars for success in overcoming distrust among foreign-born underserved populations.
Crawshaw et al. (2018) [34]	Infectious disease testing of UK-bound refugees: A population-based, cross-sectional study	Foreign-born refugees, *n* = 18,418 (48.8% female, 51.2% male)	UK	To analyse and describe data on the prevalence of all infectious diseases (tuberculosis (TB), HIV, syphilis, hepatitis B and hepatitis C) from a large cohort of refugees who underwent comprehensive pre-entry health assessments as part of the UK resettlement programme. Compare the recorded prevalence against published estimates to assess whether moving to risk-based testing would be feasible. Primary aim of the UK programme and health assessments is to facilitate early integration and linkage of the refugee to appropriate health and social services in the UK.	22 International Organization for Migration (IOM) clinics were enrolled in 14 countries. All information was entered into the medical module of IOM’s electronic database system, the Migrant Management Operational System Application (MiMOSA), which has a set of data validation rules in place, and further data validation was done by the IOM medical department using statistical and database functions.	Pre-entry health assessment; Structural; Clinical health-service.	HBV screening.	Of 188 cases of HBV, 130 (69%) were male and 132 (70%) were aged between 25 and 49 years. The overall testing yield for HBV was 2.04% (1.77–2.35%) and ranged by nationality from 0.58% (0.19–1.79%) for Iraq to 12.50% (5.24–26.96%) for South Sudan.	This study found higher diagnostic yields than expected for several diseases, including HBV. The UK programme is particularly focused on the resettlement of vulnerable refugees, and whilst the possibility of testing bias cannot be ruled out, it is likely this refugee population significantly differs from the general population of the respective country.
Dang et al. (2016) [35]	Increasing hepatitis B testing and linkage to care of foreign-born Asians, Sacramento, California, 2012-2013	Foreign-born Chinese, Hmong, Korean and Vietnamese. *n* = 1004 (62.7% female, 37.7% male)	US	To test at least 1000 foreign-born adult Asian Americans who had not been previously serologically tested for HBV. To counsel at least 90% of the people testing positive for HBsAg.	Engaged organisations linked to Chinese, Hmong, Korean and Vietnamese communities to co-sponsor HBV screenings of Asian Americans, post-test counselling and linkage to care.	Outreach testing; Individual; Community (student-run medical clinics, heritage associations and churches).	HBV screening uptake.	76 of 1004 participants tested positive for HBV and post-test counselling was provided to 51 participants.	Collaborating with community groups and addressing barriers to screening highlighted how community partners can work together to address linguistic, cultural and transportation barriers among an Asian population to implement a successful HBV initiative.
Flanagan et al. (2019) [36]	Case finding and therapy for chronic viral hepatitis in primary care (HepFREE): A cluster: randomised controlled trial	Migrants from high-risk countries. *n* = 90,250 (52% female, 48% male)	UK	To determine whether incentivising and supporting primary-care physicians in areas with a high density of migrants increases the numbers of adult migrants screened for viral hepatitis.	General practices were randomly assigned to an opportunistic screening (control) group or one of four targeted screening (interventional) groups; standard (ie, hospital-based) care and a standard invitation letter; standard care and an enhanced invitation letter; community care and a standard invitation letter; or community care and an enhanced invitation letter. In control screening, general practitioners (GPs) were given a teaching session on viral hepatitis and were asked to test all registered migrants. In the intervention, GPs were paid a nominal sum for setting up searches of records, reimbursed for signed consent forms, and supported by a dedicated clinician. Patients who were eligible for testing and tested positive for viral hepatitis in the intervention groups were eligible to enrol in a second embedded trial of community versus hospital-based care.	Incentivising GPs; Individual; Primary care (general practices).	HBV screening uptake.	Compared to control groups where only 1.7% of eligible participants took up screening, 19.5% of eligible participants in intervention groups took up screening. More than 80% of HBsAg positive participants were linked to care.	Screening migrants for viral hepatitis in primary care is effective if doctors are incentivised and supported.
Hargreaves et al. (2020) [37]	Delivering multi-disease screening to migrants for latent TB and blood-borne T viruses in an emergency department setting: A feasibility study	Foreign-born economic migrants, asylum seekers and refugees. *n* = 96 (48% female, 52% male)	UK	To investigate the delivery of an opportunistic screening model offering new migrants multi- disease screening using a one-stop blood test for latent tuberculosis infection (LTBI) combined with HIV and hepatitis B/C in an emergency department setting.	The PROMOTE study was conducted at St. Mary's Hospital Emergency Department, London, UK, which is a high-migrant area representative of many of the Boroughs across London, and where 49.8% of the resident population was born abroad. Migrant patients meeting the study inclusion criteria were offered combined infection screening in addition to the standard care they received and followed up according to routine care pathways. The screening intervention was a single venesection to test for: (i) LTBI using interferon gamma release assay (QuantiFERON-TB Gold in-tube); (ii) HIV (HIV screening assay); and (iii) hepatitis B surface antigen test (HBsAg) and (iv) hepatitis C antibody test (anti-HCVAb). Participants completed a questionnaire (piloted in this setting) with questions pertaining to time in the UK, nationality, registration with a local primary-care provider, and whether patients had previously been offered any kind of screening since their arrival. They then provided a peripheral venous blood sample, which was obtained by the research nurse.	Opportunistic screening; Individual; Other clinical health service-based (emergency department).	HBV screening uptake.	Of 96 migrants screened, there were 2 cases of HBV (1 case of HBV/LTBI co-infection).	Major gaps in current screening provision to new migrants. Need to promote screening for a more diverse range of key infections in the UK. Feasible to engage migrants in multi-disease screening.
Hsu et al. (2013) [38]	Electronic messages increase hepatitis B screening in at-risk Asian American patients: A randomized, controlled trial	Chinese and Vietnamese patients. *n* = 175 (61.4% female, 38.6% male) in intervention group	US	To determine the effectiveness of electronic health record (EHR) prompts to increase the ordering of HBV tests among primary care providers (PCPs) within an academic health system.	Providers were randomized to either receive an EHR prompt for HBV testing prior to patients’ appointments or usual care. Primary outcomes were the proportion of patients (1) whose physician ordered a HBsAg test and (2) who completed testing. Secondary outcomes were (A) test results and (B) whether the physicians followed up on the results. Providers in the intervention received an electronic prompt 24 hours before their patient’s scheduled appointment that identified the patient as a candidate for HBV testing and urged the provider to evaluate the patient for testing. The message, sent by a hepatologist to the provider’s EHR inbox, consisted of several components including a description of the Centre for Disease Control’s recommendations for HBV testing in at-risk Asian populations, the high prevalence of HBV in Asia, and a list of the appropriate laboratory tests for proper HBV screening.	EHR prompts; Individual; Primary care.	Ordering of HBV tests and linkage to care uptake.	HBsAg tests were ordered for 36/88 (40.9%) of the intervention patients and 1/87 (1.1%) of the control patients. 30 intervention patients (34.1%) and no control patients completed the HBsAg test. From the 30 intervention patients, four (13.3%) of the completed tests were HBsAg-positive, 14 (46.7%) were immune, and 12 (40%) were unprotected from HBV. Two HBsAg-positive patients were referred to specialists, and 3 unprotected patients were vaccinated for HBV.	EHR-based provider prompts significantly increased HBV testing in Chinese and Vietnamese patients.
Kelly et al. (2020) [39]	Improving uptake of hepatitis B and hepatitis C testing in South Asian migrants in community and faith settings using educational interventions – A prospective descriptive study	South Asian migrants. *n* = 219 (53% female, 47% male)	UK	To analyse the feasibility of recruiting South Asian migrants to view an educational film on CVH and the effectiveness of the film in promoting testing and increasing knowledge of CVH, and the methodological issues relevant to scale-up to a randomized controlled trial.	Qualitative data were used to inform the development of a short film to explain CVH, modes of transmission, and how to access testing. South Asian migrants were recruited to view the film in community venues (primary care, religious, community), with dried blood spot CVH testing offered immediately afterwards. Pre/post-film questionnaires assessed the effectiveness of the intervention.	Educational film; Community; Community, religious centres and primary care.	CVH testing uptake.	This study satisfied the major pre-defined criteria for success: 84% versus 40% of participants watching the film being tested. A particular success was the recruitment of the older age group and females (53%) to the film screening. A number of participants (*n* = 45) requested testing for CVH without having seen the intervention. This may suggest that other factors including community endorsement may be relevant in testing.	This study demonstrated the feasibility of recruiting first-generation migrants to view a community-based educational film promoting CVH testing in this higher-risk group, confirming the value of developing interventions to facilitate the global World Health Organization plan for targeted case finding and elimination, and a future randomized controlled trial. The study highlighted the importance of culturally relevant interventions including faith and culturally sensitive settings, which appear to minimize logistical issues and effectively engage minority groups, allowing ease of access to individuals ‘at risk’.
Navarro et al. (2014) [40]	Lower than expected hepatitis B virus infection prevalence among first generation Koreans in the US: Results of HBV screening in the Southern Californian Empire	First-generation Koreans. *n* = 1007 (60.5% female, 39.5% male)	US	To screen adult individuals to establish HBV serological diagnoses, educate, and establish linkage to care for vaccination and consideration of treatment.	10 community-screening events across 9 Korean churches over 3.6 months. Advertisements highlighting the importance of HBV testing and details were placed in Korean newspapers and newsletters several weeks prior to each event to target non-church attendees. Flyers were also posted at churches and local primary health clinics predominantly serving Koreans. A bilingual Korean nurse led the events. Screening was preceded by a brief lecture on HBV. Educational materials on HBV were distributed to all attendees. Subjects completed a brief self-administered questionnaire and provided contact information for result notification. A letter describing test results and their interpretation along with medical recommendations was mailed to each participant.	Educational and outreach intervention led by bilingual nurse; Individual; Community (churches).	HBV screening uptake and linkage to care uptake.	Prevalence of HBV was 3.0% (29 participants). Despite this barrier, 2 of the 10 infected women and 6 of the 19 men had effective follow-up visits, for a total of 27.5% (8) success in linkage to care. Of the 8 participants seen in clinic half did not require treatment. The other half was provided guidance on patient medication assistance programs and started antiretroviral therapy. The overall rate of incorrect report of immunization status was 35.1% (342), differing across education levels (*p* = 0.008) but not between genders (*p* = 0.888)	Results highlight the importance of complete serological screening among migrants before recommending immunization, as well as serological follow up to confirm successful immunization. With as many as 70% of Koreans in the US attending religious services regularly, most being first-generation immigrants, it is believed that religious congregations are therefore optimal locations for health screenings of this type.
Perumalswami et al. (2013) [41]	Hepatitis Outreach Network: A practical strategy for hepatitis screening with linkage to care in foreign-born communities	Foreign-born from countries with a high prevalence of CVH. *n* = 1603 (50.6% female, 49.4% male)	US	To provide education, screening and linkage to care in communities with a high prevalence of CVH.	Hepatitis Outreach Network combined the expertise and resources of the Mount Sinai School of Medicine, the NYC Department of Health and Mental Hygiene, and community-based organizations, to provide education, screening and linkage to care in communities with a high prevalence of CVH. Comprehensive HBV and HCV screening identifies infected patients, who then receive further evaluation from either local or Mount Sinai physicians, combined with patient navigators who organize follow-up visits.	Educational and outreach intervention; Individual; Community (public schools, places of worship, YMCA’s, public parks, hotels, business centres and train stations).	HBV and HCV screening and linkage to care uptake.	Of the 76 persons (out of 1603) with HBV infection, 43 (57%) received a medical evaluation (31 with local providers and 12 at Mount Sinai). Of the 43 HBV-infected persons evaluated, treatment was recommended and begun in 5.	Successful recruitment into screening of foreign-born persons at high risk of HBV and/or HCV may be due to the engagement of community-based organisations and by employing languages of country of origin in publicizing the events. Non-traditional venues were chosen by partnering ommunity based organizations based on their knowledge and expertise of their community. Non-traditional venues included places that community members frequented often and trusted.
Raines-Milenkov et al. (2021) [42]	Hepatitis B virus awareness, infection, and screening multi-ethnic community intervention for foreign-born populations	Refugee immigrant population. *n* = 1069 (80% female, 20% male)	US	To assess HBV awareness, vaccination status at enrolment, infection and screening among a multi-ethnic, primarily refugee immigrant population enrolled in a community-based cancer prevention intervention.	Trained bilingual and bicultural community health workers (CHWs) representative of 24 different countries of origin provided outreach, enrolment, education and navigation services. Participants were recruited from apartment complexes, faith-based locations, through community leaders and events, word-of-mouth, resettlement agencies and to a lesser degree, clinics that serve the population. CHWs navigated HBV positive participants to health care when accepted by the participants. Screening is thus an opportunity to educate and provide treatment to individuals that test positive for HBV.	Educational and outreach intervention by bicultural CHWs; Individual; Community (churches, mosques, civic groups) and primary care.	HBV screening uptake.	38% of participants accepted HBV screening after enrolment in the Building Bridges Initiative (BBI) program. More than three-quarters (76%) of those screened reported having never had HBV screening in the past. The HBV positivity rate was 6% among study participants who were screened through the BBI program. More individuals were screened in community settings rather than scheduled for clinics (68% vs 32%).	CHWs have a critical role to link HBV positive individuals to care. Despite several barriers to care being eliminated in this project, some participants with HBV declined and opted to use traditional medicine from their home country to treat their infection.
Shankar et al. (2016) [43]	A novel collaborative community-based hepatitis B screening and linkage to care program for African immigrants	African-born persons. *n* = 955 (24.5% female, 75.5% male)	US	To estimate the prevalence of current HBV infection among African-born NYC residents. To test the effectiveness of a community-based screening and linkage to care program that relies on culturally targeted patient navigators. Assess the role of known risk factors, and identify previously unknown risk factors in this population.	Hepatitis Outreach Network (HONE) partnered with a non-profit organisation to provide targeted screening for HBV to any African born adult >18 years of age. Before screening, participants were provided with a questionnaire to assess demographics and risk factors. Community members were invited to attend the screening events at no cost through public service announcements on local African radio stations, flyers at community venues, announcements by community and religious leaders, and word of mouth by the patient navigator at community events. A trained phlebotomist collected a blood sample on-site for serological testing for each patient. A culturally targeted, multilingual (English-, Arabic-, and French-speaking) professional patient navigator attempted to contact persons with their results via telephone 1 week after their screening. Upon completion of their visit, all persons were provided a round-trip public transportation fare card to cover travel costs and a $20 incentive for attending the visit.	Outreach testing and linkage to care with a culturally targeted patient navigator; Individual; Community (community centres, places of worship and sites of employment).	HBV screening and linkage to care uptake.	Of 955 persons screened for HBV, 919 had no history of liver disease, of whom 88 had a current HBV infection and 679 had exposure. 97% of infected persons were linked to care and 11 were recommended for treatment, of whom 9 started therapy.	Culturally targeted patient navigators are critical to establishing effective community-based viral hepatitis screening and linkage-to-care programs, particularly in programs targeting foreign-born persons.
Standford et al. (2016) [44]	Community-engaged strategies to promote hepatitis B testing and linkage to care in immigrants of Florida	Foreign-born nationals. *n* = 1516 (50.4% female, 49.6% male)	US	To increase the proportion of persons who are aware of their HBV status among individuals born in countries with intermediate or high prevalence of HBV infection; to increase the proportion who receive counselling and are linked to treatment and prevention services.	Community-based HBV screening programs occurred in an urban locale in both clinical and community settings. Screening, testing, and case identification activities included providing foreign-born nationals (FBNs) with an HBV risk assessment and no cost HBV test. FBNs were offered the opportunity to participate in a clinical or community setting. Communication barriers were reduced by translating program materials (i.e., written screening consent, educational materials, and test result notification letters) into 13 different languages. Participants received culturally appropriate education and awareness materials and instructions on receiving post-test counselling and results, and a $15 gift card participation incentive. Outreach post-test counselling events were conducted in conveniently located community settings. Low risk test results were mailed with risk reduction educational materials post-test. More intensive contact attempts were made for participants with high-risk test results, and participants with HBsAg positive results. Participants with high-risk test results were contacted by phone to schedule an in-person appointment to provide test results and conduct post-test counselling. HBsAg positive participants were linked to care and vaccination services accordingly.	Educational and outreach intervention by CHWs and certified medical assistants; Individual; Community (faith-based organisations, refugee servicing organisations), primary care and emergency department.	HBV screening uptake and linkage to care uptake.	1516 individuals underwent HBV testing and screening, 831 individuals received post-test counselling and, 43 HBsAg positive participants were linked to medical care.	Community-engaged strategies to improve HBV testing and service linkage in FBN populations should be considered when planning programs.
Vedio et al. (2013) [45]	Hepatitis B: Report of prevalence and access to healthcare among Chinese residents in Sheffield, UK	Chinese residents. *n* = 229 (57.5 female, 42.5% male)	UK	To facilitate HBV testing using straightforward methods in culturally appropriate settings to assess the acceptance of this testing method by Chinese residents. To estimate the local rate of undiagnosed infection and to provide access to expert healthcare for those affected.	Identified locations that would be familiar to Chinese members of the community to facilitate testing. Dried blood spot samples were collected from 229 Chinese subjects and tested for HBV and HCV infection.	Educational and outreach intervention; Individual; Community (Kinhon Chinese Centre).	HBV screening uptake and participant feedback.	28 of 229 patients had evidence of past HBV infection. 20 of 229 were diagnosed with HBsAg. 5 of the participants (4 females and 1 male, age range 23-43 years) were of Chinese origin but were born in the UK, but results were negative for all HBV markers in these 5 participants. Self-reported diagnosis showed that women had the highest rate of having had a previous diagnosis of HBV infection compared to men (5:1).	The study demonstrated the importance of outreach and targeted testing.
Xiao et al. (2021) [46]	Assessing the feasibility, acceptability and impacts of an education program on hepatitis B testing uptake among ethnic Chinese in Australia: Results of a randomised controlled pilot study	Individuals of Chinese ethnicity. *n* = 54 (69% female, 31% male)	Australia	To explore approaches to increase HBV testing in Australia’s Chinese community and inform evaluation planning, specifically to i) assess the feasibility and acceptability of HBV educational programs, and ii) compare HBV testing uptake in people receiving a tailored education resource focussing on liver cancer prevention compared with a standard HBV education package.	People of Chinese ethnicity and unsure of their HBV infection or immunity status were recruited from 10 community sites. Participants were randomised to receive an education package (comprised of a leaflet and in-person one-on-one educational session) with a focus on either 1) standard HBV-related information, or 2) liver cancer prevention. Participants completed a baseline questionnaire prior to receiving the intervention and were followed up at 6 months for a questionnaire and an opt-in semi-structured interview.	Educational and outreach intervention; Individual; Community-based organisations.	HBV testing uptake, intervention acceptability and feasibility of the study.	54 participants received an education package; baseline and follow-up data from 33 (61%) were available. The study procedures of recruitment and retention were feasible; the acceptability of the education program was moderate with improved HBV-related knowledge observed. 4 participants self-reported being tested: 1 (1/15, 7%) in the standard HBV information group and 3 (3/18, 17%) in the liver cancer prevention information group.	A larger study is required to determine if a liver cancer prevention message would improve HBV testing uptake in the Chinese community than a standard HBV education message. Support from healthcare providers, community-based testing programs, and public health education programs are likely needed to motivate diagnostic testing among Chinese people at risk of HBV infection.
Zibrik et al. (2018) [47]	Let’s Talk About B: Barriers to hepatitis B screening and vaccination among Asian and South Asian immigrants in British Columbia	Korean, Chinese, Filipino and South Asian Immigrants. *n* = 827 (70% female, 30% male)	Canada	To describe different types of viral hepatitis and the severity of infection. Emphasize HBV prevention including screening, vaccination and risk reduction strategies. Outline symptoms and transmission routes of HBV. Describe HBV treatment and pathway for care; identify community resources and supports for people living with HBV Promote a healthy lifestyle to prevent the progression of liver disease.	Culturally tailored HBV education workshops were delivered over 12 months. Data from pre- and post-workshop surveys and 2-week and 1-month follow-up interviews were collected and analysed to evaluate knowledge gaps and challenges around HBV prevention and screening. Barriers, health care service gaps and facilitators identified in the interviews were coded and analysed.	Culturally tailored education workshops; Community; Community (community centres, immigration settlement service centres, organised health events and religious/cultural gathering places).	Self-reported action related to HBV prevention and management.	Data were collected from 827 participants who attended workshops. 633 participants took part in the 2-week and 1-month follow-up interviews conducted via telephone. Of these, 55% took specific action related to HBV prevention or management such as proactively sought out/read information (online or in print) on HBV; talked to their doctor/health professional about HBV testing/care; got tested for HBV following the workshop; scheduled an appointment to see their doctor/health professional; checked their vaccination status or that of a family member; got vaccinated against HBV.	Study findings support the need for culturally tailored HBV public education and outreach programs to further advance HBV immunization and awareness in British Columbia. Addressing barriers and developing targeted programmatic strategies identified in this study will promote more effective HBV education programming and improve uptake of HBV screening and vaccination in British Columbia’s immigrant populations.
